# Calcium ions open a selectivity filter gate during activation of the MthK potassium channel

**DOI:** 10.1038/ncomms9342

**Published:** 2015-09-23

**Authors:** David J. Posson, Radda Rusinova, Olaf S. Andersen, Crina M. Nimigean

**Affiliations:** 1 Department of Anesthesiology, Weill Cornell Medical College, 1300 York Avenue, New York, New York 10021, USA; 2 Department of Physiology and Biophysics, Weill Cornell Medical College, 1300 York Avenue, New York, New York 10021, USA; 3 Department of Biochemistry, Weill Cornell Medical College, 1300 York Avenue, New York, New York 10021, USA

## Abstract

Ion channel opening and closing are fundamental to cellular signalling and homeostasis. Gates that control K^+^ channel activity were found both at an intracellular pore constriction and within the selectivity filter near the extracellular side but the specific location of the gate that opens Ca^2+^-activated K^+^ channels has remained elusive. Using the *Methanobacterium thermoautotrophicum* homologue (MthK) and a stopped-flow fluorometric assay for fast channel activation, we show that intracellular quaternary ammonium blockers bind to closed MthK channels. Since the blockers are known to bind inside a central channel cavity, past the intracellular entryway, the gate must be within the selectivity filter. Furthermore, the blockers access the closed channel slower than the open channel, suggesting that the intracellular entryway narrows upon pore closure, without preventing access of either the blockers or the smaller K^+^. Thus, Ca^2+^-dependent gating in MthK occurs at the selectivity filter with coupled movement of the intracellular helices.

## Abstract (web summary)

Ion channels open and close to allow the regulated passage of ions through the membrane. Here the authors use selective ion channel blockers to analyse this regulation in a potassium channel and show that the gate is in the selectivity filter, past the entrance to the channel.

## Graphical abstract

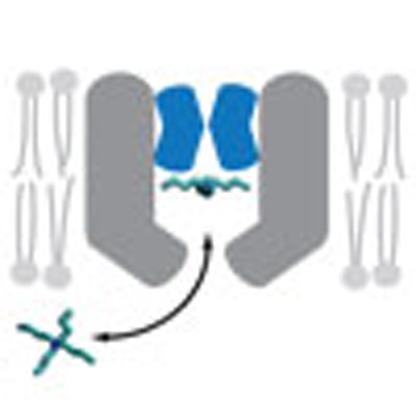

Calcium ions regulate diverse cellular processes including synaptic transmission, muscle contraction, exocytosis, gene transcription and cell motility^1^. These complex processes are controlled by Ca^2+^ channels, as well as proteins that respond to increases in cytosolic Ca^2+^, including Ca^2+^-dependent ion channels^2^. The ubiquitously expressed large conductance voltage and Ca^2+^-activated K^+^ (BK) channel, for example, binds Ca^2+^ to a cytosolic domain called a gating ring, resulting in large increases in K^+^ conductance that hyperpolarizes the cell membrane and decreases electrical excitability^3^. By coupling membrane excitability to intracellular Ca^2+^ concentrations, BK channels serve as key elements in the regulation of Ca^2+^-dependent cellular processes. To understand the molecular events underlying normal or pathophysiological Ca^2+^ signalling, it is necessary to clarify how Ca^2+^ binding opens BK channels.

The BK channel (Slo1) is a member of the eukaryotic Slo family of K^+^ channels^4^ that evolved from prokaryotic K^+^ channels, such as the MthK channel from *Methanobacterium thermoautotrophicum*, which is similarly activated by cytosolic Ca^2+^ (Fig. 1a)^5^,^6^,^7^,^8^. The ability to express and purify large quantities of MthK for functional and structural analyses has advanced MthK as a model for Ca^2+^-dependent gating in BK channels. X-ray crystallography studies show that the cytosolic Ca^2+^-binding gating rings for the BK and MthK channels are structurally related^9^,^10^,^11^ (Fig. 1a) and undergo similar Ca^2+^-dependent conformational changes as shown by crystal structures of these domains in the presence and absence of calcium^12^. In contrast to the BK channel, however, structural models are available for the isolated MthK pore domain^13^ and full-length MthK channel^5^, both in a putative open conformation, facilitating the interpretation of structure–function relationships in channel gating (Fig. 1a). (There is no structure of the MthK channel pore in a putative closed conformation.) In this context, it is important that MthK channels lack the voltage-sensor domains that confer voltage dependence on BK channels, thereby isolating the mechanism for Ca^2+^-dependent gating and simplifying experimental determination of the gating mechanism in MthK compared with BK. Understanding how the MthK pore opens and closes in response to Ca^2+^ thus provides a solid context for interpretation of gating studies of eukaryotic Slo channels, including BK.

Two gate locations that control ion permeation have been proposed in K^+^ channels—a structural constriction at the intracellular side of the pore, called a ‘bundle crossing’, and a structurally undefined closing of the selectivity filter^14^. A large aqueous vestibule that lies between these two possible gate locations forms a binding site for quaternary ammonium (QA) channel blockers, which have provided a tool for probing the gating mechanism^15^,^16^. In the case of voltage-gated channels, numerous functional studies have shown that activation gating involves the opening of an intracellular gate^14^,^17^,^18^. Alternatively, several types of ligand-gated channels, including BK channels^19^,^20^,^21^,^22^, have been proposed to employ a different activation gating mechanism that involves changes at the selectivity filter^23^,^24^,^25^,^26^,^27^,^28^. However, the studies in support of selectivity filter gating in BK channels are not definitive due to the complexity of BK channel gating (voltage- and Ca^2+^-dependent gating) combined with the use of voltage-dependent blockers, leading to uncertainty in identification of the gate location.

Subsequent structural studies of several prokaryotic channels, employing various channel mutations, have provided molecular models of open and closed-channel states^29^,^30^,^31^,^32^,^33^,^34^,^35^, strengthening the notion that many channels employ an intracellular bundle-crossing gate. Although only the open-state structure of the MthK pore is available, it was proposed that the closed channel has an intracellular bundle-crossing gate similar to KcsA, based on the sequence homology between MthK and KcsA in the pore region^36^. In a previous study^37^, we showed that membrane voltage controls a selectivity filter gate and not a bundle-crossing gate in MthK, but it was not determined whether this gate is also controlled by Ca^2+^.

Here we investigate the mechanism of Ca^2+^-dependent gating using purified MthK channels reconstituted into lipid bilayers of defined composition. We show that Ca^2+^-dependent gating occurs at the selectivity filter, and not at the bundle crossing, by detecting closed-channel block by large QA molecules that bind immediately below the selectivity filter. By utilizing a stopped-flow-based flux assay^38^,^39^, Ca^2+^ and blocker are very quickly applied to MthK liposomes with the time resolution essential for discriminating between gate locations. Tetrapentylammonium (TPeA) binds ∼100-fold slower to the closed state than to the open state, indicating a significant conformational change during closing that reduces but does not eliminate blocker access. These results suggest that hydrated potassium ions, which are smaller, also have rapid access to the aqueous vestibule beneath the selectivity filter. We propose that Ca^2+^ binding to the gating ring may induce an expansion at the intracellular channel entryway that leads to opening of the primary conduction gate at the selectivity filter. Such a mechanism may be conserved in the Slo K^+^ channel family, including the BK channel.

## Results

### Experimental concept

**Figure 1:**
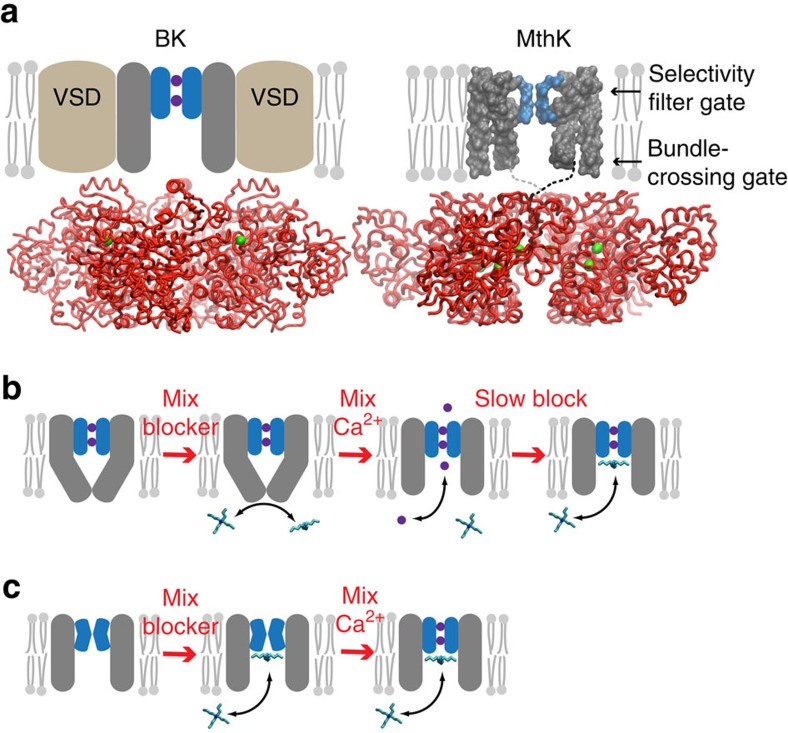
Blockers as probes for Ca^2+^-dependent gate location in K^+^ channels. (**a**) Comparison of BK (left) and MthK (right) channel architectures. The gating rings (red ribbons) with bound Ca^2+^ (green spheres) are structurally conserved between BK (pdb 3U6N) and MthK (pdb ILNQ). The unknown transmembrane structure for BK is illustrated with voltage-sensor domains (VSDs, brown) and pore domain (grey) with selectivity filter (blue). MthK lacks VSDs and the full-length protein structure is available, except for linkers between pore and gating ring (dashed lines). Locations of two putative gates are indicated by arrows. Only two subunits for the transmembrane domains are illustrated for clarity. (**b**) The gated access model: the pore domain has a bundle-crossing gate that prevents QA blocker binding to the aqueous vestibule. Rapid activation by Ca^2+^ results in immediate channel activity (purple circles and arrow) followed by blocker binding just below the selectivity filter. (**c**) The selectivity filter gate model: blocker has access to the binding site in both closed and open channels and steady-state block is reached before MthK is activated by Ca^2+^.

To probe whether activation of MthK channels by Ca^2+^ occurs by opening a bundle-crossing or a selectivity filter gate (Fig. 1a), we examined the state dependence of channel block by QA ions that bind between the two candidate gate locations, immediately below the selectivity filter^16^,^37^,^40^,^41^. If MthK has a bundle-crossing gate closure (Fig. 1b), QA blockers do not have access to the binding site within the pore when the channels are closed. Alternatively, if MthK channels have a selectivity filter gate, QA blockers may access the binding site when the channels are closed, in a size-dependent manner (Fig. 1c). Thus, if QA blockers bind within closed MthK channels, gating must occur above the binding site, implicating the selectivity filter as the Ca^2+^-dependent gate.

To investigate whether closed channels can be blocked, we incubated closed MthK channels (in the absence of Ca^2+^) with blocker, then rapidly activated the channels with Ca^2+^ and measured the channel activity. If the gate is at a bundle crossing, blockers do not have access to the channel in the closed state and rapid opening with Ca^2+^ will result in maximal channel activity followed by slower blocker binding within the pore and a subsequent decrease in activity (Fig. 1b). This sequence of events was observed in studies of voltage-dependent K^+^ (Kv) channels, demonstrating gated blocker access in those channels^15^,^42^. If the Ca^2+^-dependent MthK gate is at the selectivity filter, blockers may access their binding site in the closed channel and rapid opening with Ca^2+^ will only show the activity of the fraction of unblocked channels (Fig. 1c).

To distinguish between these two models, several conditions have to be met. First, to measure MthK block by QA molecules in the closed state, we need to assess the channel activity immediately following activation by Ca^2+^; both Ca^2+^ application and the activity measurement must be carried out with high time resolution. Second, the rate of channel activation by Ca^2+^ must be faster than the rate of QA blocker binding. Third, MthK channels must be closed in the absence of Ca^2+^.

**Figure 2:**
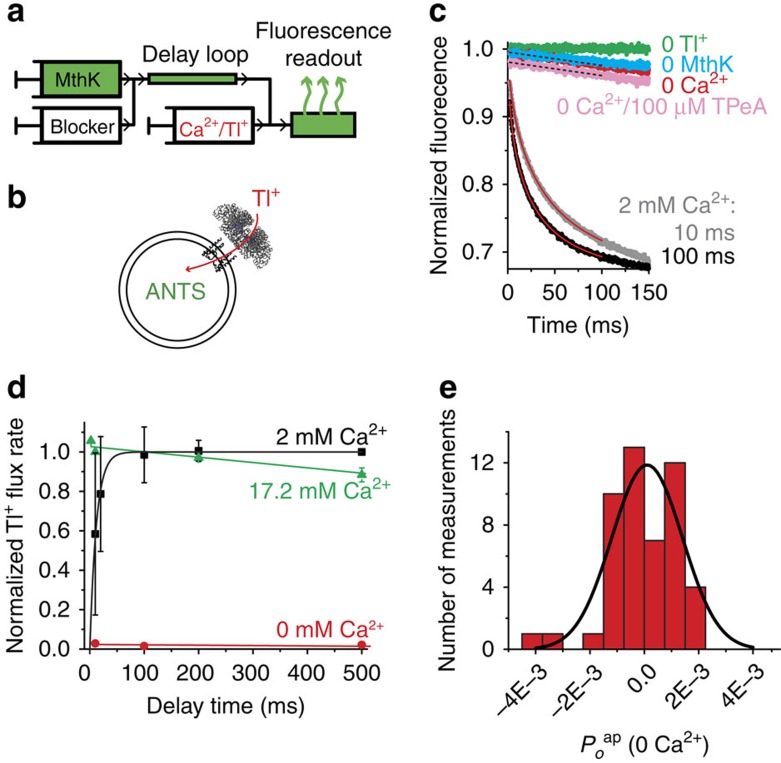
MthK is closed in 0 Ca^2+^ and is activated quickly with Ca^2+^. (**a**) Schematic representation of the sequential-mixing stopped-flow device. The mixing sequence for a closed-state block assay is shown from left to right (Supplementary Fig. 1). MthK-reconstituted liposomes are mixed with blocker and incubated in a delay loop for a defined time interval followed by mixing with activating Ca^2+^ and fluorescence-quenching Tl^+^ into an optical cell for fluorescence readout. (**b**) Open MthK channels allow Tl^+^ entry (red arrow) into the liposomes, quenching the fluorescence of the encapsulated ANTS dye. (**c**) Fluorescence quench curves for MthK liposomes after 10 or 100 ms (grey and black, respectively) incubation with 2 mM Ca^2+^. Flux rates were from fits to stretched exponentials (red lines). Control fluorescence is in the absence of Tl^+^ (green). A small leak of Tl^+^ into liposomes is observed in experiments without Ca^2+^ (red), similar to the leak in MthK-free liposomes (cyan). The non-specific Tl^+^ leak in MthK liposomes was also measured in the presence of 100 μM TPeA (pink). A linear fit was used to analyse the slow Tl^+^ leak signals (black dotted lines). (**d**) Relative Tl^+^ flux rates as a function of Ca^2+^ incubation time for 0 (red), 2 (black) and 17.2 mM (green) Ca^2+^. Symbols are the mean±s.d. from three (or two for 0 mM Ca^2+^)-independent measurements. (**e**) Histogram of 49 independent estimates of apparent open probability in the absence of Ca^2+^ (*P*_o_^ap^(0 *Ca*^2+^), calculated using equations (4, 5, 6, 7, 8) in the Methods section). The average value was −0.00004±0.0002 (mean±s.e.m.). The histogram was fit with a Gaussian distribution (black line) with mean at *P*_o_^ap^(0 *Ca*^2+^)=0.0001±0.0003 (0.01±0.03%) and s.d. *σ*=0.0013±0.0003.

We satisfied the first condition by using a stopped-flow instrument with millisecond time resolution to rapidly mix MthK-reconstituted liposomes with Ca^2+^ and Tl^+^ (Fig. 2a; Supplementary Fig. 1). Tl^+^ enters the liposomes through open MthK channels and quenches an internal fluorescent dye, 8-aminonaphthalene-1,3,6-trisulfonic acid (ANTS; Fig. 2b). Thus, the initial rate of fluorescence quenching is a measure of the channel activity, obtained only 2 ms following the application of Ca^2+^ (the instrument dead time) by fitting the fluorescence decline to a stretched exponential function (Methods; Equation 1) and calculating the Tl^+^ flux rate at 2 ms from this fit (Methods; equation 2).

### Ca^2+^ rapidly activates MthK

The second condition necessary to measure closed-state channel block is that MthK activation has to be fast. We measured the rate of MthK activation by incubating MthK liposomes with several Ca^2+^ concentrations in the delay loop and then mix with Tl^+^ (Supplementary Fig. 1a). As expected, longer incubations with Ca^2+^ resulted in faster fluorescence quenching (increased channel activity; Fig. 2c). At 2 mM Ca^2+^, the time course of MthK activation was fit with a single-exponential function with a time constant of 12.1±0.4 ms (Fig. 2d, black squares; Equation 3). To achieve an even faster activation rate, we increased the [Ca^2+^] to 17.2 mM where full activity was reached during the mixing dead time, indicating that the activation time constant is in the microsecond regime, faster than we can measure with our time resolution (Fig. 2d, green). Thus, using 17.2 mM Ca^2+^, we could ‘instantaneously’ activate MthK channels and meet our second experimental criterion. We also observed that after a few seconds MthK channel activity declined (Supplementary Fig. 2), similar to what was previously reported using electrophysiological recordings with MthK from *Escherichia coli* spheroplasts^43^, but not observed in steady-state single-channel recordings in lipid bilayers^6^,^7^,^8^. This process did not interfere with our blocker studies, which were performed within a few hundred millisecond of channel activation (see below).

### Open probability of MthK in the absence of Ca^2+^

To satisfy the third requirement for measuring closed-state channel block, we estimated the open probability of MthK channels under our experimental conditions in the absence of Ca^2+^. A non-zero open probability in the absence of Ca^2+^ would result in blocker binding to the fraction of channels that are open, which would diminish our ability to discriminate between the gated access and state-independent access models. Channel openings in zero Ca^2+^ are undetected in most single-channel recordings in lipid bilayers^6^,^8^ and we expected a similar result using our flux assay.

In the absence of Ca^2+^, very slow fluorescence decays are observed for MthK liposomes, similar to those recorded in protein-free controls, suggesting that the channel activity in the absence of Ca^2+^ is extremely low, as expected from previous results using single-channel recording (Fig. 2c). Thus, most of this Tl^+^ influx in zero Ca^2+^ is due to non-specific Tl^+^ leak across the liposomal membrane^38^. To quantify such low channel activity, we determined the flux contribution, if any, arising from rare MthK openings, by subtracting the non-specific leak obtained in the presence of saturating concentrations of channel blocker, which is a measure of the true membrane ‘leak’ because any flux through the channels is now blocked. Since the result is a difference between two very small numbers, we performed a statistical analysis of 49 experimental estimates of this very small influx rate (Fig. 2e). This analysis showed that the residual ion permeability through MthK channels in the absence of Ca^2+^ is statistically indistinguishable from zero (Fig. 2e; Supplementary Note).

### TPeA blocks closed MthK channels with slow kinetics

**Figure 3:**
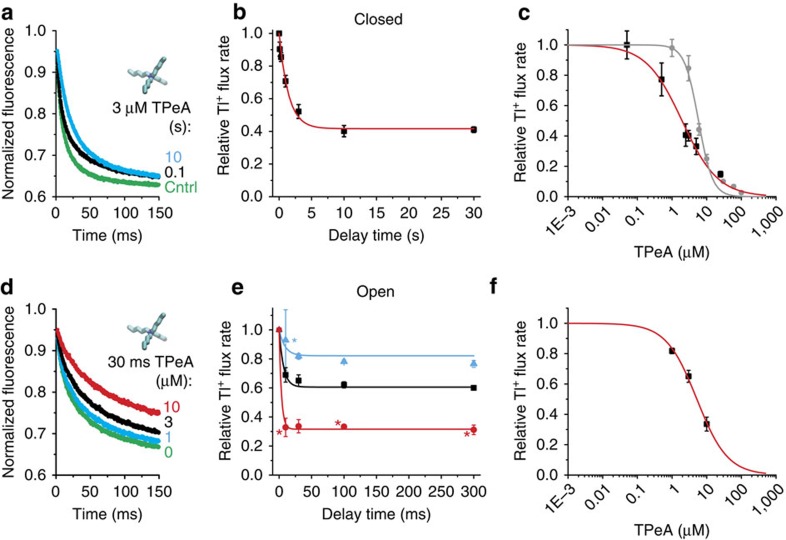
TPeA blocks closed and open MthK. (**a**) Fluorescence quench traces after closed-state incubation with 3 μM TPeA for 0.1 (black), 10 s (cyan) and no blocker control (green) (average of 4–7 repeats). (**b**) Relative Tl^+^ flux rates versus incubation time from data as in **a**. Red line is a fit with equations (10 and 11) (*τ*_eq_=1.6±0.2 s, 
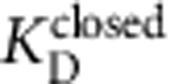
=2.1±0.2 μM, 
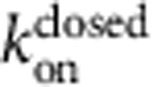
=0.14±0.02 μM^−1^ s^−1^). (**c**) Dose–response curve for closed-state TPeA equilibrium block after 10-s blocker incubation. Red line is a Hill equation fit (Equation 12, 
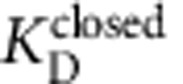
=2.0±0.2 μM, *n*_H_=0.84±0.08). Dose–response curve for open-state block after the 2 ms mixing time (grey circles). The grey line has no theoretical meaning. (**d**) Fluorescence quench traces after 30-ms incubation with 17.2 mM Ca^2+^ to measure open-state block by 0 (green), 1 (cyan), 3 (black) or 10 μM (red) TPeA (average of 4–7 repeats). (**e**) Relative Tl^+^ flux rates versus incubation time with open MthK for three [TPeA], coloured as in **d**. The results were simultaneously fit to equation (11) (lines; 
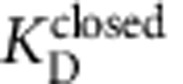
=4.6±0.2 μM, *k*_on_^closed^=20±3 μM^−1^ s^−1^). (**f**) Dose–response curve for TPeA binding to open MthK after 30-ms incubation with blocker (results from **e**) was fit by the Hill equation (red line; 
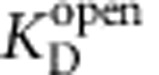
=5.2±0.3 μM, *n*_H_=0.98±0.07). Mean±s.d. from at least three independent samples, except for experiments marked (*) in **e** where *n*=2.

With all of the necessary experimental conditions met, we addressed our central question; do QA blockers bind to closed channels? Blocker binding to closed MthK would indicate that the channels lack a bundle-crossing gate and must close at the selectivity filter. For this, we incubated MthK channels with TPeA in the absence of Ca^2+^ followed by very rapid channel activation to detect whether the blocker had bound to the closed channels (Fig. 2a). The fluorescence decay was slower for longer blocker incubation periods, suggesting that TPeA was indeed able to bind to and block closed MthK channels (Fig. 3a). Short incubations with TPeA (∼100 ms) resulted in little inhibition, whereas incubations for >1 s led to substantial inhibition of channel activity (Fig. 3b). A single-exponential fit to these results yields an equilibration time constant of ∼2 s (Fig. 3b, red line; Equation 10) from which we extracted blocker kinetics (
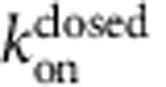
=0.14±0.02 μM^−1^ s^−1^) and affinity (
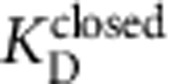
=2.1±0.2 μM) of TPeA to closed MthK (Fig. 3b; Equation 11; see also Table 1).

To further characterize closed MthK block, we measured the TPeA dose–response curve by equilibrating the closed channel for 10 s with various blocker concentrations and recording channel activity (Fig. 3c, red). A fit using the Hill (Equation 12) provided another estimate of the apparent closed-state dissociation constant, 
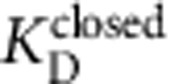
=2.0±0.2 μM, similar to what we found above (Fig. 3b), and a Hill coefficient of 0.84±0.08 (Fig. 3c, blue dotted line), which is <1, the predicted Hill coefficient for a bimolecular reaction model for blocker binding to MthK (Equation 12). It is possible that the TPeA dose response is shallower than expected because our measurement of closed-state block requires opening the channel. If the blocker affinity changes after the channel opens, then at higher blocker concentrations, where the blocker equilibration rate is faster, the blockers may partially equilibrate to open channels within our instrument dead time, thus contaminating our closed-channel block measurements. To determine whether this is the case, we measured the blocker binding to the open state.

### TPeA blocks open MthK channels with fast kinetics

To investigate TPeA block of open MthK channels, we used a sequential-mixing protocol in which MthK liposomes were first incubated with Ca^2+^ and blocker before assaying channel activity (Supplementary Fig. 1b). As expected, the fluorescence decay was slower as the blocker concentration was increased (Fig. 3d). In addition, steady-state block was reached in ∼30 ms (Fig. 3e), which was faster than expected given our previous determinations using single-channel block in lipid bilayer recordings^37^. We estimated the open-state apparent blocker dissociation constant and kinetics by globally fitting equation (11) to the equilibration time course for three different blocker concentrations (Fig. 3e, lines). The fitted values for the apparent open-state dissociation and on-rate constants were 
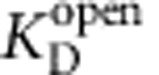
=4.6±0.2 μM, higher than for closed MthK, and 
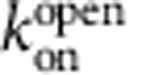
=20±3 μM^−1^ s^−1^, much faster than for closed MthK (for direct comparison, see Table 1), confirming that TPeA blocks MthK in a state-dependent manner and that open-state blocker kinetics is fast. We also plotted the TPeA dose–response curve for the results using 30-ms incubation with TPeA, which resulted in another estimate (Equation 12) of 
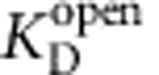
=5.2±0.3 μM with a unitary Hill coefficient, as expected for a bimolecular interaction between blocker and open MthK (Fig. 3f).

The fast TPeA open-state kinetics and the state dependence of TPeA block suggest that our closed-state block measurements may indeed reflect an additional component of re-equilibration towards open-state block during the mixing dead time. To evaluate the degree of ‘contamination’, we designed an experiment to measure directly the amount of blocker binding to the MthK open state that occurs during this time. To do this, we simultaneously mixed MthK liposomes with TPeA, high Ca^2+^ and Tl^+^ (Supplementary Fig. 1d) and immediately measured the activity so that only the amount of open-state TPeA block occurring during the mixing dead time was measured (Fig. 3c, grey). TPeA concentrations below ∼6 μM did not result in significant decreases in the measured Tl^+^ flux rate (Fig. 3c, grey symbols) and the apparent closed-state inhibition measured at these concentrations (Fig. 3c, red symbols) reflects mostly the degree of closed-state block. Consequently, our closed-state blocker equilibration measurements using 3 μM TPeA (Fig. 3b) should accurately reflect the affinity and kinetics for closed-state block. The apparent closed-state inhibition by TPeA concentrations at ∼6 μM and higher reflect a mixture of closed- and open-state block, with rapid blocker re-equilibration after channel opening (overlap of grey and red symbols in Fig. 3c).

### A model for state-dependent MthK block by TPeA

We refined our estimates of the thermodynamic and kinetic parameters for closed-state block by analysing the TPeA dose–response curve in Fig. 3c using a state-dependent model that explicitly accounted for blocker re-equilibration during stopped-flow mixing. As expected, because at 3 μM TPeA there was little blocker re-equilibration to the open state (Fig. 3b), using this model led to minor adjustments of the TPeA closed-state block parameters (Supplementary Note). Specifically, the estimates of 
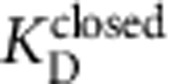
, 
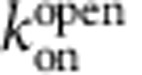
 and 
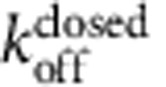
 were lowered by small amounts: ∼40% (from 2.1 to 1.3 μM), ∼20% (from 0.14 to 0.11 μM^−1^ s^−1^) and ∼50% (from 0.29 to 0.14 s^−1^), respectively (compare Table 1 with Supplementary Table). The model also fits well the sub-unitary slope of the TPeA dose–response curve (Supplementary Fig. 3) by incorporating the blocker re-equilibration, which was significant at concentrations higher than ∼6 μM (Fig. 3c, red and grey symbols).

These results provide a model for both closed- and open-state MthK channel block with a modest state dependence in blocker affinity and a large state dependence in blocker kinetics (Table 1). This suggests that TPeA encounters a steric obstacle or energy barrier before reaching its binding site in the closed channel. To explore this possibility, we investigated the kinetics of closed-state binding for two other QA blockers, bromobenzyltributylammonium (Br-bTBA) and *N*-(4-[benzoyl]benzyl)-*N*,*N*,*N*-tributylammonium (bbTBA). These blockers have benzyl-containing groups that are less flexible than alkyl chains and may increase the blocker hydrodynamic radius. bbTBA in particular is larger than TPeA and is thus predicted to encounter greater hindrance to binding inside the closed channel.

### Larger QA molecules block closed MthK with slower kinetics

**Figure 4:**
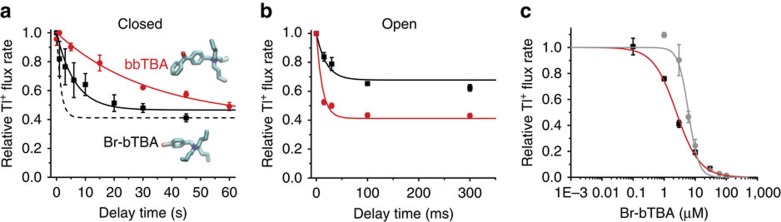
Br-bTBA and bbTBA block closed MthK more slowly than TPeA. (**a**) Relative Tl^+^ flux rates as a function of Br-bTBA and bbTBA (structures inset) incubation with closed MthK channels. Blocker equilibrations were fit to exponential functions, *τ*_Br-bTBA_=9±3 s, *τ*_bbTBA_=32±7 s (Equation 10, black and red solid lines, respectively). The fitted blocker time course for TPeA (from Fig. 3b) is shown for comparison (black dashed line). (**b**) Relative Tl^+^ flux rates versus incubation time with open MthK for 1 (black squares) and 3 μM (red circles) bbTBA. The results were simultaneously fit to equation (11) (lines; 
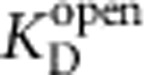
=2.1±0.2 μM, 
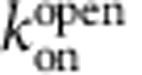
=17±2 μM^−1^ s^−1^). (**c**) Closed-state Br-bTBA dose–response curve for 45-s blocker incubations before activation of MthK by 17.2 mM Ca^2+^ (black squares). The results were fit to the Hill equation (red line; 
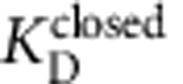
=2.5±0.2, *n*_H_=1.16±0.09). Dose response for open-state Br-bTBA block after the mixing dead time (grey circles). Grey line has no theoretical meaning. Symbols are the mean±s.d. from at least three (only two for bbTBA data in **b**) independent measurements.

**Figure 5:**
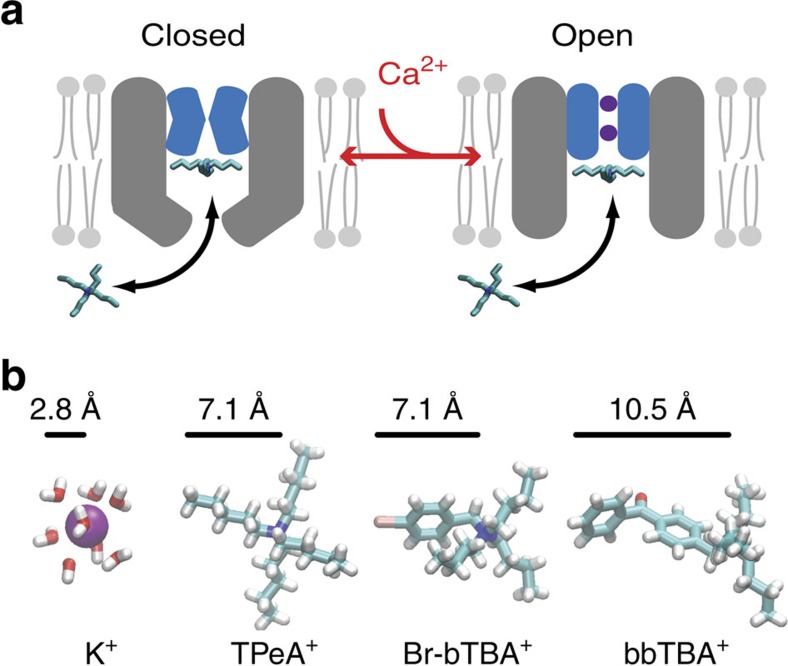
Closure at the MthK selectivity filter is accompanied by a conformational change near the intracellular entryway. (**a**) Model for QA block of the closed and open MthK channel. The selectivity filter is non-conductive in the absence of Ca^2+^ and the intracellular entryway (grey) is narrowed but still allows blocker/K^+^ entry into vestibule (left). Channel activation opens the selectivity filter gate (purple K^+^ inside blue filter) and increases blocker access rate into the pore (right). (**b**) The radius of a hydrated K^+^ is smaller than the extended structures in TPeA^+^, Br-bTBA^+^ and bbTBA^+^, suggesting that K^+^ can access the closed MthK intracellular entryway despite the constriction.

Br-bTBA and bbTBA are QA blockers with either a single brominated benzyl group or two benzyl groups, respectively, attached to the central nitrogen (Fig. 4a, structure insets, Fig. 5b). Using our flux assay, we found that 3 μM Br-bTBA blocked ∼50% when applied to closed MthK channels, similar to TPeA, while only 1 μM bbTBA was sufficient to block the same amount, indicating higher affinity for the closed MthK. Unlike TPeA, however, Br-bTBA equilibration required tens of seconds, while the larger bbTBA requires approximately six times as long, as illustrated by the closed-state blocking time courses (Fig. 4a, black squares and red circles). The apparent on-rates estimated from these results show that Br-bTBA and bbTBA bind approximately fivefold and eightfold slower than TPeA (Table 1), consistent with the larger, more rigid aromatic ring structures that may increase the hydrodynamic radii relative to TPeA (Fig. 5b).

Because bbTBA block of closed channels was much slower than TPeA block, we measured the kinetics of open-channel block by equilibrating 1 and 3 μM bbTBA with Ca^2+^-activated MthK channels (Fig. 4b). The results indicate that bbTBA binds to open MthK as quickly (
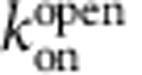
=17±2 μM^−1^ s^−1^) as TPeA (Table 1). We also evaluated whether the closed-state block parameters for Br-bTBA are ‘contaminated’ by open-state block re-equilibration, as observed for TPeA (Fig. 3c). We tested whether Br-bTBA equilibrates rapidly with the open state by applying the blocker simultaneously with Ca^2+^ and Tl^+^ to measure the amount of block occurring during the 2 ms mixing time (Fig. 4c, grey circles). At 3 μM Br-bTBA, the open-state block during the mixing time was very low, indicating that our measurement for the closed-state block at this concentration (Fig. 4c, black squares) is very little affected, similar to what we found for TPeA (compare Figs 3c and 4c).

We also measured the Br-bTBA open-state block parameters using single-channel bilayer recordings and found that the affinity and on-rate are almost identical to those of TPeA and bbTBA^37^, indicating that the three blockers have similar fast access to the MthK open state (Supplementary Fig. 4), whereas they have much slower and different access rates to the closed state (Fig. 4a). This suggests that the entryway to the MthK aqueous vestibule narrows on channel closure.

## Discussion

The gating mechanisms in eukaryotic Ca^2+^- and voltage-gated BK channels have been explored using blockers^19^,^21^,^44^,^45^,^46^,^47^,^48^,^49^ and thiol-modifying probes^20^,^22^ to assess conformational changes within the pore. Taken together, most of these studies are suggestive of a gating mechanism ‘deeper’ inside the pore than for K_v_ channels^50^, though a molecular-gating model has yet to be firmly established^51^. In particular, the complexity of the BK channel, namely, the confounding interplay of voltage-dependent gating, Ca^2+^-dependent gating and voltage-dependent block has hindered a definitive demonstration that BK gating occurs at the selectivity filter. Here we elucidated the mechanism of Ca^2+^ dependent gating in MthK, a prokaryotic BK channel homologue with a conserved Ca^2+^-dependent gating ring but devoid of voltage-sensing domains^5^, thus allowing unhampered study of the Ca^2+^-dependent gate. Using a flux assay with high time resolution^38^,^39^, we were able to characterize the closed-state block of MthK by QA blockers (Fig. 1), and find that Ca^2+^ induces changes at the intracellular channel entryway, which controls K^+^ conductance via a gate at the selectivity filter.

We found that TPeA blocks MthK with state-dependent kinetics, suggesting a conformational change near the bottom of the pore in the closed state. One possible change that could lead to slower block of the closed state is a reduction in the size of the intracellular entryway (Fig. 5a). We examined this possibility further using Br-bTBA and bbTBA, two QA molecules with benzene-containing groups that are more rigid and in the case of bbTBA longer than the pentyl groups in TPeA (Fig. 5b). Br-bTBA and bbTBA bind approximately fivefold and eightfold slower to the closed state than TPeA, respectively (comparing *k*_on_ values in Table 1), consistent with the hypothesis that there is a steric barrier slowing the entry of the QA blockers into the channel vestibule in the closed state (Fig. 5a). The slower access rate suggests that the radius of the entryway to the closed state is comparable to the radii of the blockers^52^,^53^. The radii of the extended blocker structures (Fig. 5b) are between 7 and 11 Å but the effective hydrodynamic radii may be different. For example, the flexibility of alkyl chains may reduce the effective size of TPeA (to about ∼4.5 Å)^54^. Thus, we can safely assume that the entrance to the closed pore is likely >5 Å in radius, about twice the hydrated radius of K^+^ (∼2.8 Å; Fig. 5b). We conclude that K^+^ should be able to readily enter the closed-channel vestibule, indicating the absence of an intracellular bundle-crossing gate for permeant ions.

Thus, the channel closure in zero Ca^2+^ that occurs at the selectivity filter is coupled with a conformational change such as a partial bundle-crossing-like movement that fails to constrict the entryway enough to prevent ion and blocker access into the pore. We previously observed a similar effect for voltage-dependent closure of MthK^37^. These conformational changes may serve as an allosteric pathway, connecting the binding of Ca^2+^ at the intracellular gating ring to the selectivity filter gate at the extracellular side of the pore. Coupling between movements at the bundle crossing and the selectivity filter has also been shown to occur in other channels such as KcsA^55^,^56^ and K_V_ channels^57^ during activation-coupled inactivation. However, in these channels, the expansion of the intracellular entryway is coupled with a closing of the selectivity filter rather than an opening as proposed here for MthK and recently for K2P channels^58^.

Recent experimental and computational studies have explored how the size of the intracellular entryway may influence the conductance of K^+^ channels. In BK channels, two glutamate residues at the entryway were systematically mutated to vary the effective electrical resistance for K^+^ entry^59^. Using tryptophan substitutions at these sites, Geng *et al.* estimated that the radius of the entryway was reduced from ∼9 Å for the wild type to ∼5 Å in the mutant. The narrow opening in the mutant BK (smaller than the size of TPeA) would be expected to slow the kinetics of block by large blockers, such as TPeA, with relatively little effect on K^+^ movement. Indeed, the outward K^+^ current in the mutant BK channel was reduced by only ∼30% (ref. 59). Furthermore, molecular dynamics simulations of a Shaker K^+^ channel^60^ and an open structure of KirBac3.1 (ref. 31) suggest that K^+^ enters the pore cavity with essentially no energy barrier when the opening is comparable to the size of a hydrated K^+^, consistent with blocker studies of inward rectifier channels where QA block is significantly slowed for blockers larger than tetrapropylammonium (∼4-Å radius)^53^. Hydrated K^+^ ions likely have rapid access into the closed MthK channel vestibule and are prevented from passing through the selectivity filter.

The state dependence of TPeA block in MthK shows that the blocker on-rate for closed channels is ∼140-fold slower than for open channels (Table 1). This fold change is similar to the state-dependent rates (∼200-fold slower) for chemical modification by MTS reagents of a residue (A313C) deep inside the BK channel pore^20^. It appears that both MthK and BK channels allow probe accessibility deep into the non-conducting channel. Although the rate of modification by MTS reagents and binding of QA blockers reflect different reactions, the similarity in state-dependent rates suggests that similar conformational changes may occur at the intracellular entryway of these homologous channels. Supporting this conclusion, mutation of a residue deep inside the pore of MthK (A88D) results in a constitutive open state^61^, similar to what was observed using mutagenesis^62^,^63^ or MTS modification^20^ of nearby residues in the BK channel. MTS modification rates and gating perturbation by mutagenesis, however, may reflect state-dependent changes of a specific side chain, whereas blocker state-dependence reports on the overall access into the cavity and is likely less sensitive to the local environment of individual side chains.

Our experiments using the liposome-based flux assay uncovered properties of MthK block that contrasted with our previous results from single-channel recording in planar lipid bilayers. First, the measured open-state affinity for both TPeA and bbTBA near 0 mV suggests the blockers bind to MthK in liposomes with a sixfold lower affinity than to MthK in planar bilayers. Second, the kinetics of block/unblock were even more sensitive to the experimental context; the TPeA kinetics were ∼50-fold faster for MthK in liposomes as compared with bilayers^37^. Since the functional properties of ion channels, such as KcsA^38^ and BK^64^, can be regulated by lipid bilayer thickness, it is possible that the greater thickness of decane-containing planar lipid bilayers^65^ may alter the intracellular entrance to the open MthK channel, thereby inducing tighter blocker binding with slower on- and off-rates. Alternatively, the differences in lipid composition or the presence of Tl^+^, a modulator of BK channel gating^66^, may influence MthK gating or QA block. Despite the increased kinetics in liposomes, bbTBA, for example, still accesses open MthK approximately sixfold slower than BK although faster than QA molecules were reported to access other K^+^ channels^52^,^53^. This suggests that MthK has a cavity similar in size to BK channels, albeit somewhat smaller^19^,^22^,^46^. Regardless of these differences, however, the mechanism of Ca^2+^ activation was not disrupted by changes in the lipid bilayer; MthK channels are completely closed in the absence of Ca^2+^ and activate with millimolar Ca^2+^ concentrations in both liposomes and bilayers.

In conclusion, the selectivity filter is the Ca^2+^-dependent gate for K^+^ permeation in MthK. This gating mechanism is markedly different from the gating mechanisms of KcsA and voltage-dependent channels, in which expansion of the intracellular entryway leads to inactivation, a closure of the selectivity filter. In MthK, however, Ca^2+^ binding elicits conformational changes of the gating ring and expansion of the intracellular pore entryway, which are coupled with opening of a selectivity filter gate. This mechanism has likely been conserved from prokaryotes to the eukaryotic Slo family of K^+^ channels, including BK.

## Methods

### MthK purification and reconstitution

MthK channels were expressed and purified as previously described^37^. Unless indicated otherwise, all reagents were obtained from Sigma-Aldrich and procedures were performed at 25 °C. *N*-(4-[benzoyl]benzyl)-*N*,*N*,*N*-tributylammonium (bbTBA) was from Spectra Group Limited Inc. and 4-bromobenzyltributylammonium bromide (Br-bTBA) was custom-made (The Chemistry Research Solution LLC). Molecular models of QA blockers were created using the program eLBOW^67^. Protein was purified in 100 mM KCl, 20 mM Tris (pH 7.6), with 5 mM *N*-decyl-β-D-maltopyranoside (Anatrace), stored for one night at 4 °C and run through a Superdex 200 gel filtration column (GE Healthcare) immediately before reconstitution into liposomes (large unilamellar vesicles). Protein reconstitution was similar to methods recently published^38^. A amount of 15 mg of 1,2-dioleoyl-sn-glycero-3-phosphocholine and 1-palmitoyl-2-oleoyl-sn-glycero-3-[phospho-rac-1-glycerol] (3:1 DOPC:POPG, Avanti Polar Lipids) were dried in a round-bottom flask under nitrogen gas and further dried overnight in a vacuum desiccator. Liposomes were prepared in 100 mM KNO_3_, 10 mM HEPES (pH 7.0), 25 mM 8-aminonaphthalene-1,3,6-trisulfonic acid and disodium salt (ANTS, Life Technologies; reconstitution buffer). Lipids were solubilized in 1.5 ml reconstitution buffer by the addition of 35 mg CHAPS (Anatrace) and sonicated to clarity using a bath sonicator (Avanti Polar Lipids). Purified MthK protein was added to the solubilized lipids at a concentration of 30 μg mg^−1^ lipid and incubated for 30 min. Liposomes were formed by detergent removal using 1 g of SM-2 Biobeads (BioRad) added with an additional 1.5 ml of reconstitution buffer and rotated in a glass tube for 2 h. The resulting liposome suspension was sonicated for 20 s in a bath sonicator (Branson) and extruded through a 100-nm-pore polycarbonate filter (Avanti Mini-Extruder). Extravesicular ANTS was removed using PD-10 desalting columns (GE Healthcare) using 140 mM KNO_3_ and 10 mM HEPES (pH 7.0) (flux buffer). For the contents-mixing assay (Supplementary Fig. 2), MthK liposomes were made as above, except that the internal solution contained either 25 mM ANTS or a membrane impermeant fluorescence quencher, 90 mM p-xylene-bis-pyridinium bromide (DPX, Life Technologies). The ANTS-containing liposomes were mixed with DPX-containing liposomes for 10 ms before mixing with pH 11.7 buffer (to give a final pH of 8.5) containing either 0 Ca^2+^ (control, black symbols) or 17.2 mM Ca^2+^ (red symbols), and the ANTS fluorescence was recorded for 10 s. If liposome fusion had occurred, the ANTS- and DPX-containing solutions would mix, resulting in fluorescence quenching. As a control, the ANTS-loaded liposomes were mixed with 90 mM DPX in the presence of 1% Triton X-100, which permeabilizes the liposomes to measure the maximum quenching response.

### Stopped-flow Tl^+^ flux assay

Tl^+^ quenches the fluorophore ANTS fluorescence and serves as a K^+^ mimic being able to permeate K^+^-conducting channels. Taking advantage of MthK permeability to Tl^+^, channel activity was monitored by the time course of ANTS quenching (Fig. 2b). MthK activity was assayed by estimating the initial Tl^+^ flux rate following rapid mixing of ANTS-loaded liposomes with a Tl^+^-containing buffer using a SX-20 stopped-flow spectrofluorometer (Applied Photophysics Leatherhead, UK) following^38^,^68^ (see Supplementary Fig. 1 for diagrams of the mixing protocols described below). Because of the unavoidable heterogeneity in liposome sizes, the fluorescence quench time course cannot be described by a single exponential, and the results between 2 (instrumental dead time) and 100 ms were fit to stretched exponential functions (Equation 1) and the rates of these fitted stretched exponential functions at 2 ms, defined as the Tl^+^ flux rates in our measurements, were calculated from fit parameters (Equation 2, derived in ref. 69) using MATLAB (MathWorks).




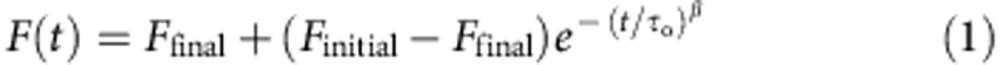







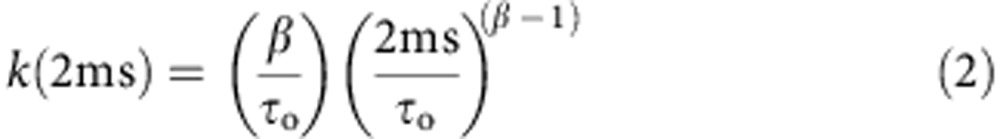




where *F*_final_ and *F*_initial_ are the final and initial fluorescence values, respectively. The empirical parameters *τ*_o_ and *β* are the time constant and the exponential-stretch parameter, respectively, and reflect the dispersity of liposome sizes as well as changes in channel activity occurring during the measurement.

To record the channel activation and block time courses, two sequential solution mixings were performed, separated by a programmed delay (≥10 ms) that occurs in the delay loop (Fig. 2a, Supplementary Fig. 1). The solutions used were as follows: premix solution—140 mM KNO_3_ and 10 mM HEPES (pH 11.7); control solution—140 mM KNO_3_ and 10 mM HEPES (pH 8.5); and quench solution—50 mM TlNO_3_, 94 mM KNO_3_ and 10 mM HEPES (pH 8.5). Initially, MthK liposomes (in flux buffer, above) were mixed 1:1 with premix solution to set the mixture pH to 8.5 in the delay loop. The sample was then mixed 1:1 with either control solution or quench solution in the optical cell for recording ANTS fluorescence. Channel was activated by the addition of 2 × [Ca^2+^] to the premix solution and 1 × [Ca^2+^] to the control and quench solutions (to account for the dilution that occurs in each mixing reaction). Closed-state channel block was studied by the addition of 2 × [blocker] to the premix solution in the absence of Ca^2+^, 1 × [blocker] and 34.4 mM Ca^2+^ to the control or quench solution. To measure activation or block on a timescale faster than 10 ms, we introduced 2 × [Ca^2+^] with or without 2 × [blocker] in the quench solution (with dead time of 2 ms) instead of through the delay loop. We observed a small systematic dilution error during the first mixing, and nulled the discrepancy between additions of blocker during mix 1 versus mix 2 by the addition of 5% higher [blocker] in the zero-delay protocol. Because MthK requires Ca^2+^ to open, only channels reconstituted with the cytoplasmic gating ring facing the extra-liposomal solution contribute to the MthK activity measurements. Individual sample-experiment combinations were repeated three to seven times, and Tl^+^ flux rate determinations were highly reproducible and averaged. Except where noted, at least three independent proteoliposome preparations were studied and the reported results denote mean±s.d.

### Data analysis and modelling

Average Tl^+^ flux rates were further analysed using Origin 6.0 (OriginLab). All rates were internally normalized to the maximum activity from experimental controls. The apparent MthK activation rate at 2 mM Ca^2+^ was estimated by fitting the time course of the Tl^+^ flux rates to a single exponential:




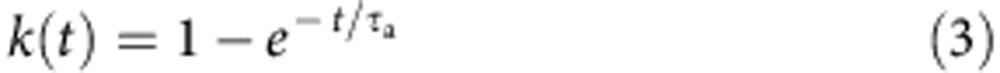




where *t* is delay time between solution mixing, *k*(*t*) is the Tl^+^ flux rate at time *t* and *τ*_a_ is the apparent time constant for channel activation.

To estimate MthK activity in nominally 0 Ca^2+^ (no Ca^2+^ buffers were used), we compared the Tl^+^ influx rates observed with liposomes in the absence of Ca^2+^ to the Tl^+^ influx rate observed in 17.2 mM Ca^2+^. Tl^+^ flux rates are very slow in 0 Ca^2+^, and we could not employ a stretched exponential fit in this case (Equation 1). Instead, we fit the data between 2 and 100 ms to a linear function (Equation 4), which determined the geometric slope of the fluorescence data, *m*, at 0 ms, defined as the activity measurement for these very slow quench traces. To normalize the low activity, slope *m*, to the maximal activity in 17.2 mM Ca^2+^, we obtained an estimation of the maximal geometric slope at 0 ms by fitting the high Ca^2+^ fluorescence quench curves to the modified stretched exponential function (Equation 5, derived in ref. 69). We used here equation (5) and not equation (1) (which was used to analyse channel activity for all other experiments) because the stretched exponential function described in equation (1) does not have a finite slope at 0 ms (equation (4) is a Taylor approximation of equation (5) for the initial Tl^+^ flux). The slope of equation (5) at 0 ms (Equation 6) was calculated from best-fit parameters to estimate the control MthK activity rate, *m*_control_.




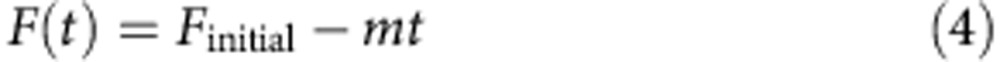







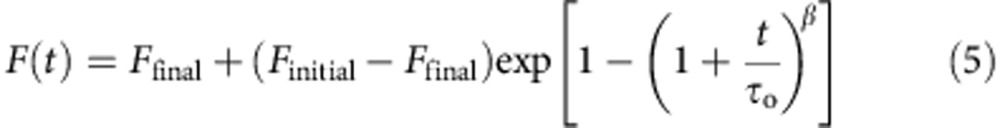







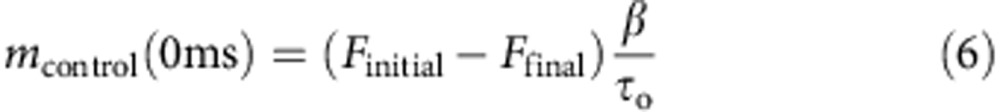




To distinguish between slow Tl^+^ entry due to non-specific movement of TlNO_3_ across liposome membranes and specific movement of Tl^+^ through MthK channels in the absence of Ca^2+^, we recorded in immediate succession the slow fluorescence decay in the absence and presence of saturating channel blocker (100 μM TPeA), yielding two activity estimates *m*_total_ and *m*_lipid_, respectively. Ion movement through the mostly closed MthK channels, *m*_channel_, then was obtained by subtracting the Tl^+^ flux across the liposomal membranes from the total Tl^+^ flux (Equation 7) and the estimated apparent MthK open probability in the absence of Ca^2+^, *P*_o_^ap^(0 Ca^2+^), was estimated by normalizing to the control activity in high Ca^2+^ (Equation 8).




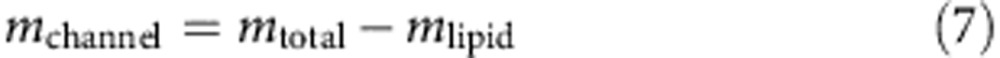







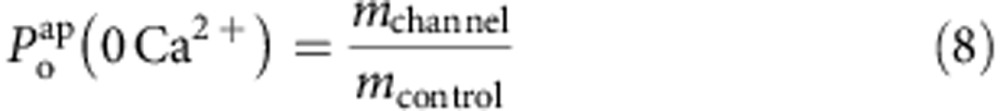




These measurements were performed 49 times (employing two independent liposome preparations) and the estimates of *P*_o_^ap^(0 Ca^2+^) were plotted in a histogram and fitted to a Gaussian distribution (Equation 9).




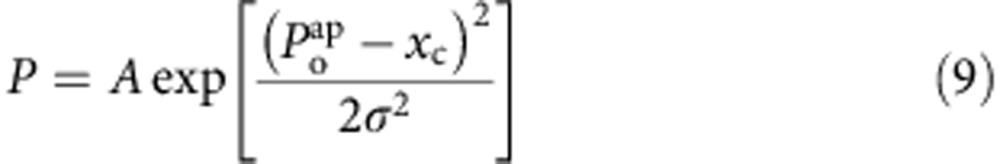




where *A* is the amplitude, *x*_c_ is the centre of the distribution and *σ* is the s.d.

Blocker equilibration time courses were fit with a single-exponential decay:




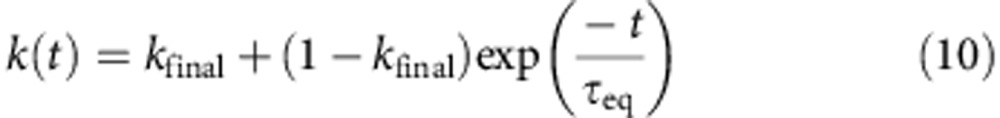




where *k*_final_ is the final Tl^+^ flux rate (after blocker equilibration) and *τ*_eq_ is the apparent blocker equilibration time constant.

To extract the blocker (*B*) affinity and kinetics, blocker equilibration to either open or closed channels (*MthK*), is modelled as a bimolecular reaction:




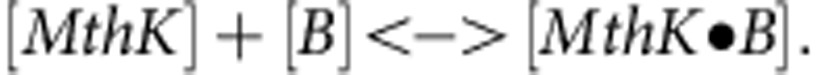




Because the blocker concentration is much larger than the channel concentration, the fraction of channels without blocker bound decays exponentially, with an equilibration time constant (Equation 10) determined by the blocker kinetics. The steady-state activity is determined by the blocker dissociation constant (Equation 11):




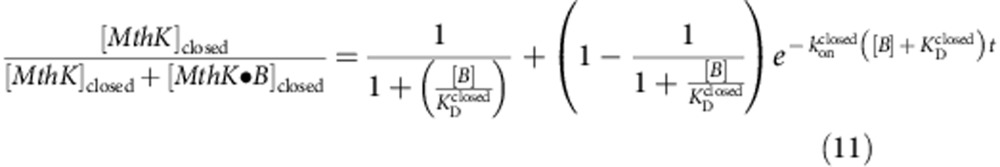




where 
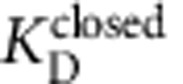
 and 
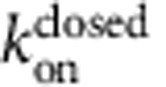
 are the closed-state blocker dissociation and on-rate constants, respectively, and [*B*] is the blocker concentration. Equilibration to the open state follows the same expression, using the parameters 
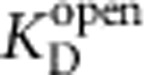
 and 
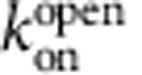
, the open-state blocker dissociation and on-rate constant, respectively.

Blocker dose–response curves were fit with the Hill equation:




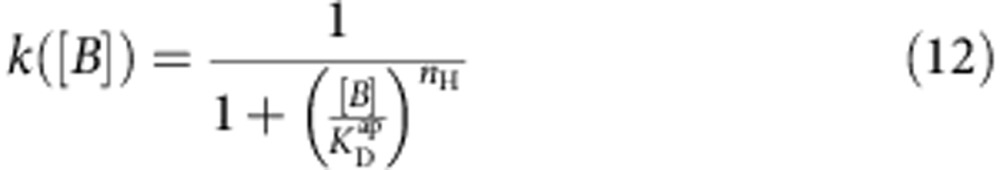




where 
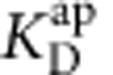
 is the apparent blocker dissociation constant, [*B*] the blocker concentration and *n*_H_ the Hill coefficient.

### Gramicidin Tl^+^ flux assay

Gramicidin (gA) was reconstituted into large unilamellar vesicles using 3:1 DOPC:POPG and DC_22:1_PC lipids using published protocols^68^ for the experiments shown in Supplementary Fig. 2b to rule out QA blockers as lipid bilayer modifiers^68^ and also Ca^2+^-induced liposome fusion as a control for Ca^2+^-induced desensitization of MthK (Supplementary Note). Fluorescence quench rates were measured as described above after exposure to Ca^2+^ or blockers for various time intervals. Small decreases in flux in the presence of Ca^2+^ are likely due to Ca^2+^ block of gA channels^70^.

### Single-channel analysis using planar lipid bilayers

Single MthK channels were recorded using Axopatch 200A (Molecular Devices) in planar lipid-decane bilayers consisting of 3:1 POPE:POPG lipids (Avanti Polar Lipids), as previously described^37^. For the experiments in Supplementary Fig. 4, channels were recorded in symmetric 200 mM K^+^ (190 mM KCl and 10 mM KOH), 10 mM HEPES (pH 8.5/HCl) and activated by *trans* 5 mM Ca^2+^ with and without 0.5 μM Br-bTBA (also in *trans*) at –100, –25 and +25 mV. Analysis was performed using QuB (www.qub.buffalo.edu) to fit closed and open dwell time distributions with an open-channel block scheme (Supplementary Fig. 4), as described^37^. Transitions between open (O) and open-blocked (OB) states were used to estimate the open-state dissociation constant *K*_d_^open^ and the voltage dependence, 
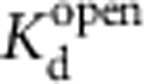
(*V*), was fit with a K^+^-coupling model, equation (13) (see ref. 37):




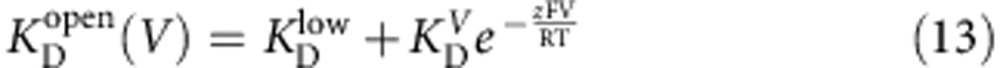




where 
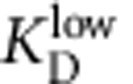
 is the maximal blocker affinity and 
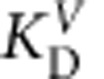
 is an affinity amplitude for the exponential term and *z* is the valence for blocker K^+^ coupling.

## Additional information

**How to cite this article:** Posson, D. J. *et al.* Calcium ions open a selectivity filter gate during activation of the MthK potassium channel. *Nat. Commun.* 6:8342 doi: 10.1038/ncomms9342 (2015).

## Supplementary Material

Supplementary Information Supplementary Figures 1-4, Supplementary Table 1, Supplementary Note 1 and Supplementary References

## Figures and Tables

**Table 1:** Apparent blocker dissociation constants and kinetics.

**Blocker**	**MthK state**	***K***_**D**_^ap^ **(μM)**	***k***_**on**_^ap^ **(μM**^−1^ **s**^−1^**)**	***k***_**off**_^ap^ **(s**^−1^)*
TPeA	Open	4.6±0.2	20±3	92±14
	Closed	2.1±0.2	0.14±0.02	0.29±0.05
bbTBA	Open	2.1±0.2	17±2	35±6
	Closed	0.7±0.2	0.018±0.002	0.012±0.004
Br-bTBA	Closed	2.6±0.4	0.028±0.005	0.07±0.02

^*^k_off_^ap^*=k*_on_^ap^ *K*_D_^ap^.
