# Supplementary material for: Calcium ions open a selectivity filter gate during activation of the MthK potassium channel
Source: Nat Commun. 2015 Sep 23;6:8342. doi: 10.1038/ncomms9342 (PMC4580985; doi:10.1038/ncomms9342)
Supplement: Supplementary Information — Supplementary Figures 1-4, Supplementary Table 1, Supplementary Note 1 and Supplementary References [file ncomms9342-s1.pdf]

Supplementary Information for "Calcium ions open a selectivity filter gate during activation of the MthK potassium channel" by David J. Posson, Radda Rusinova, Olaf S. Andersen, Crina M. Nimigean.

Supplementary Figure 1.

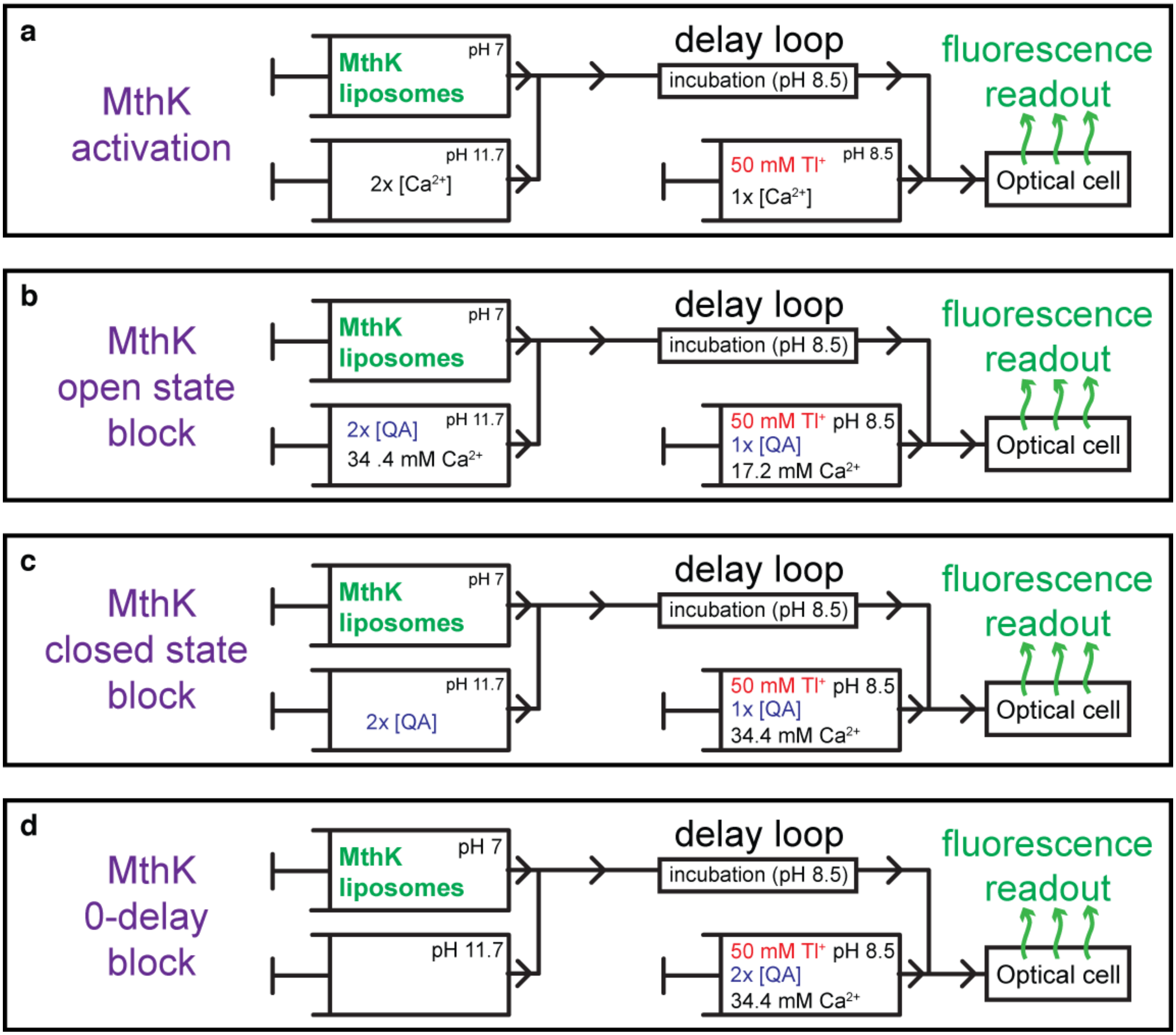

Supplementary Figure 1. Diagrammatic illustration of our stopped-flow mixing protocols. (**a**) Time course of MthK activation: Mix MthK LUVs with Ca^{2+}, and change the pH to 8.5, incubate for a period of time (in the delay loop), and then mix with Tl^{+} in the optical cell. (**b**) Time course of MthK open-state block: Mix MthK LUVs with high Ca^{2+} (17.2 mM final concentration) and QA blocker, and change the pH to 8.5, incubate for a period of time, and then mix with Tl^{+} in the optical cell. (**c**) Time course of MthK closed-state block: Mix MthK LUVs with QA blocker (no Ca^{2+}), and change the pH to 8.5, incubate for a period of time, and then mix with high Ca^{2+} (17.2 mM final concentration) plus Tl^{+} in the optical cell. (**d**) Measurement of the amount of open state block developing during channel fast activation with Ca^{2+} during the 2 ms dead time (0-delay block): Mix MthK LUVs with the pH 11.7 buffer (to reach a final pH of 8.5), incubate for 100 ms, and then mix with a solution containing QA blocker, high Ca^{2+} (17.2 mM final concentration) plus Tl^{+} in the optical cell.

Supplementary Figure 2.

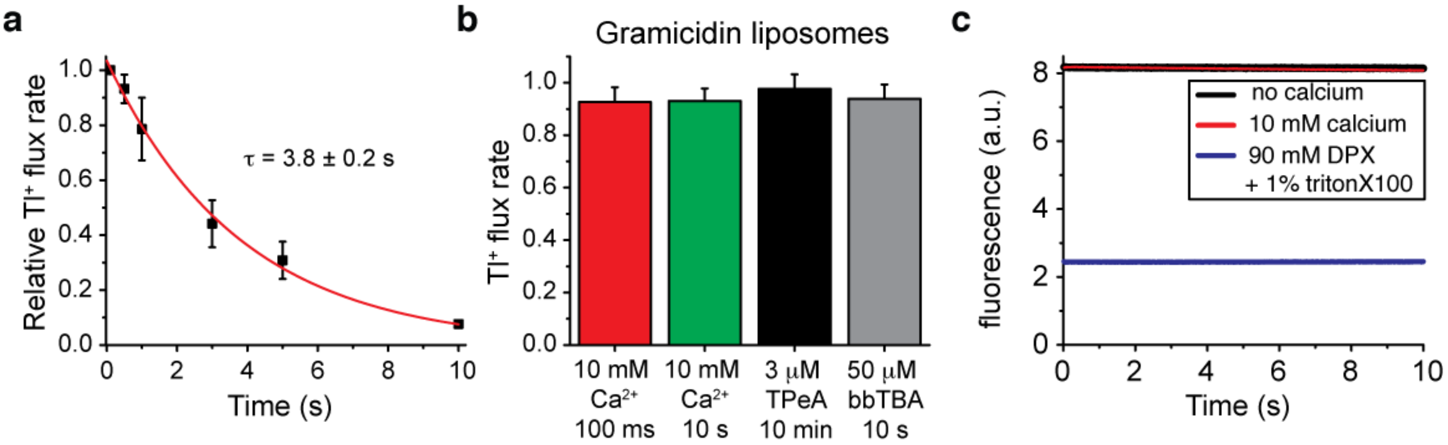

Supplementary Figure 2. Apparent desensitization of MthK activity in the presence of Ca^{2+}. (**a**) Experiments were performed as in Fig. 2, with 17.2 mM Ca^{2+} incubation times longer than 1 s prior to mixing Tl^{+} and measuring the fluorescence quench rate (black symbols). The results were fit to a single exponential decay with $\tau = 3.8 \pm 0.2$ s. Desensitization is nearly complete by 10 s. (**b**) The red and green bars corresponding to fluorescence quench rates using gramicidin (gA) LUVs were measured after exposure to 10 mM Ca^{2+} for 100 ms (red bar) or 10 s (green bar). The small reduction in flux rate, compared to the no-Ca^{2+} control, is not time-dependent, in stark contrast to the loss of channel activity in MthK LUVs in **a**. The small decrease in flux is likely due to Ca^{2+} block of the gA channels. The black and grey bars depict fluorescence quench rates, also through gA LUVs, exposed to 3 μM TPeA for 10 min or 50 μM bbTBA (N-(4-[benzoyl]benzyl)-N,N,N-tributylammonium) for 10 s, respectively. The blockers do not alter the gA activity, indicating that they do not alter bilayer properties (or induce liposome fusion). All rates are normalized to control values, no Ca^{2+} (for red and green) or no blocker (black and grey). All LUVs are made of 3:1 DOPC:POPG except for the TPeA experiment (black bar), which was performed using $DC_{22:1}PC$ LUVs. (**c**) A contents-mixing assay also provides no evidence for proteoliposome fusion in high Ca^{2+}. The ANTS-containing MthK LUVs were mixed with DPX-containing MthK LUVs for 10 ms before mixing with pH 11.7 buffer (to give a final pH of 8.5) containing either 0 Ca^{2+} (control, black symbols) or 17.2 mM Ca^{2+} (red symbols), and the ANTS fluorescence was recorded for 10 s. No quenching (meaning no contents mixing) was observed over the 10 s incubation. As a control, the ANTS-loaded LUVs were mixed with 90 mM DPX in the presence of 1% Triton X-100, which permeabilizes the liposomes and shows the maximum quenching response (blue symbols).

Supplementary Figure 3.

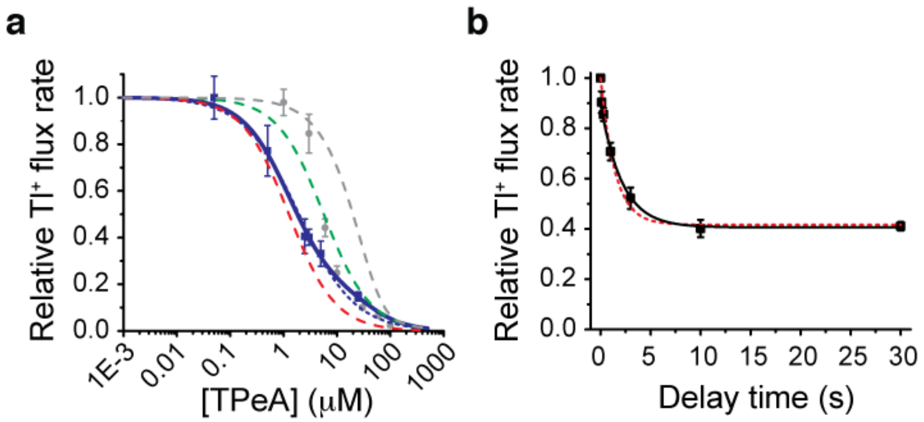

Supplementary Figure 3. Analysis of closed state TPeA block using a state-dependent model. (**a**) The dose response curve for closed state TPeA block results from Fig. 3**c** (blue squares) was fit to the state-dependent model described in Supplementary Note (blue line, Eq. S3). The fitted parameters were: $K_D{}^{closed}$ = 1.3 ± 0.2 μM, $K_D{}^{open}$ = 5.7 ± 0.9 μM and were used to simulate the red and green dashed lines, which are the theoretical dose response curves for the closed and open state block, respectively. The dose response curve for the amount of block occurring during stopped flow mixing (grey symbols also from Fig. 3**c**) is shown for comparison with the theoretical curve (grey dashed line, Eq. S5) using the fitted values for $K_D{}^{open}$ = 5.7 μM (from the state-dependent model) and $k_{on}{}^{open}$ = 20 μM^{-1} s^{-1} (Fig. 3**e**, constrained parameter in the state-dependent model). (**b**) The closed state 3 μM TPeA block equilibration results from Fig. 3**b** were fit to the state-dependent model (black solid line, Eq. S4). The fitted parameter was: $k_{on}{}^{closed}$ = 0.11 ± 0.02 μM^{-1} s^{-1}. The dotted red line is the single exponential fit from Fig. 3**b** for comparison.

Supplementary Figure 4.

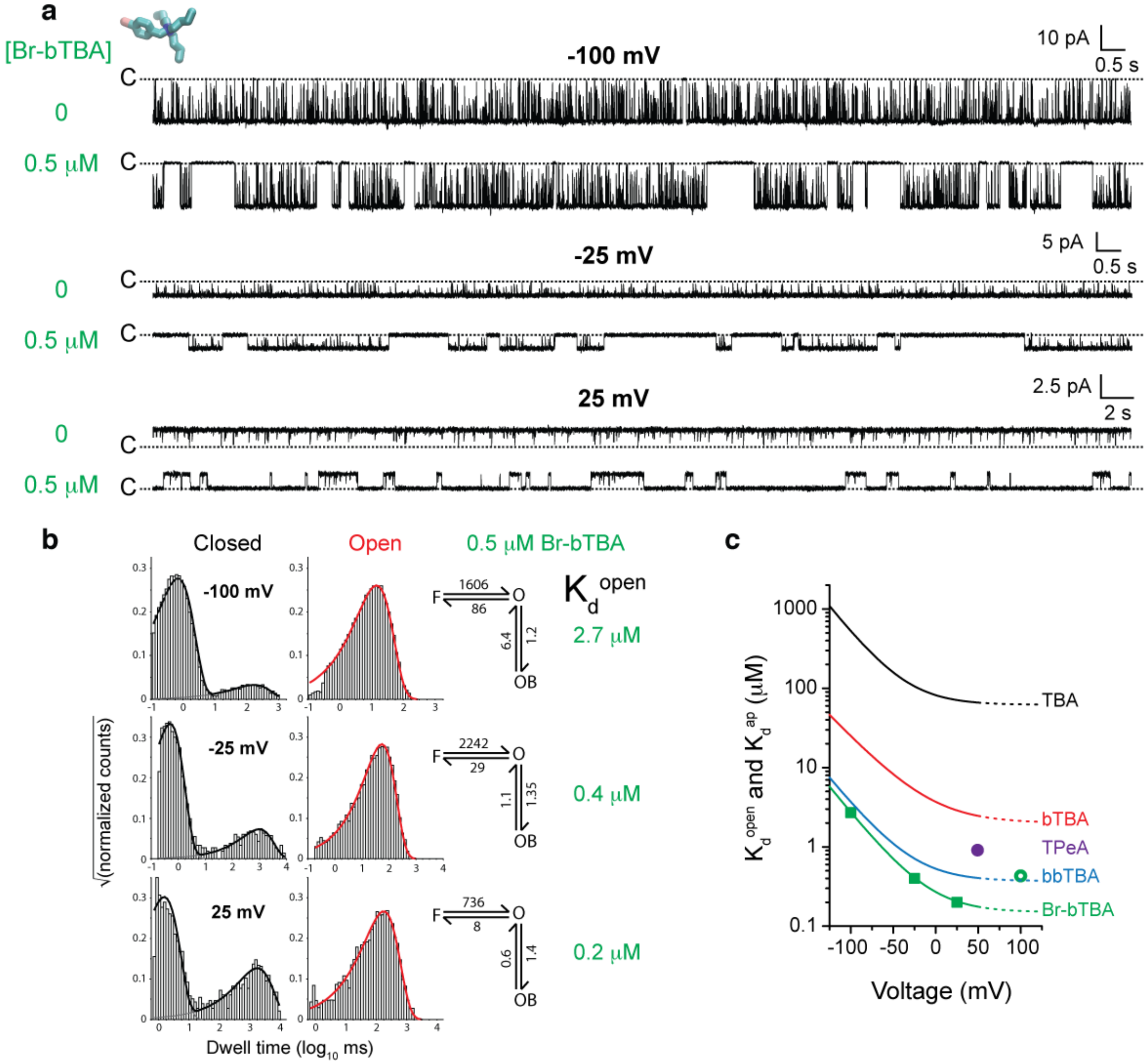

Supplementary Figure 4. Open state MthK block by Br-bTBA. (**a**) Single channel recordings of MthK activated by 5 mM Ca^{2+} in planar lipid bilayers (symmetric 200 mM K^+, pH 8.5) with and without 0.5 μM Br-bTBA at −100 mV, −25 mV, and +25 mV. (Note the different scale bars for the three voltages.) (**b**) Closed and open dwell time distributions for the recordings in **a**. The distributions were fit to an open-channel block scheme (shown to the right of the distributions) where the transition between O and OB represents blocker binding to the open state, and F (called 'flicker') is a blocker-independent brief closed state. The numbers in the schemes are rate constants (in s^{-1}) related to the exponential components fitted to the distributions (black and red lines). The rate constants describing transitions between states O and OB (in s^{-1}) were used to estimate K_d^{open} (in μM). (**c**) The voltage dependence for K_d^{open} from **b** (green square symbols) was fit with a K^+-coupling model (green line, Eq. 13): K_d^{open} = 0.15 μM + 0.12 μM exp(-0.79 V/25.69), where V is the membrane voltage (in mV). The dotted lines are extrapolations of K_d^{open} above 50 mV. Previous results obtained with tetrabutylammonium (TBA), benzyl-tributylammonium (bTBA), N-(4-[benzoyl]benzyl)-N,N,N-tributylammonium (bbTBA) (black, red, and cyan lines, respectively) and TPeA (purple circle) are shown for comparison, taken from Posson et al.[1] The apparent K_d of Br-bTBA block at 100 mV, determined by the percent block compared with control (green open circle), indicates that the blocker affinity is reduced for the closed MthK channels compared with the extrapolated K_d^{open} (green dotted line), similar to what was previously found for bTBA and bbTBA[1].

Supplementary Table. State-dependent model for TPeA block of the MthK channel.

**Channel State**	K_D **(μM)**	k_{on} **(μM^{-1} s^{-1})**	*k_{off} **(s^{-1})**
Closed (0 Ca^{2+})	1.3 ± 0.2	0.11 ± 0.02	0.14 ± 0.03
Open (17.2 mM Ca^{2+})	5.7 ± 0.9	20 ± 3	114 ± 25

*$k_{off} = k_{on} K_D$

Supplementary Note

MthK channels desensitize in the presence of Ca^{2+}.

The proteoliposome-based Tl^+ flux assay uncovered a property of MthK gating that contrasted with our previous results obtained using single-channel recording in planar lipid bilayers. When we incubated MthK LUVs with Ca^{2+} for more than a few hundred milliseconds, we observed an exponential decrease in fluorescence quench rates (Supplementary Fig. 2**a**). This apparent MthK desensitization, not observed in planar lipid bilayer recordings[2-4] but reported for patch-clamp experiments of MthK in *E. coli* spheroplasts[5], results in near complete loss of channel activity after ~10 s. Such a decrease in fluorescence quench rates could occur for many reasons, including Ca^{2+}-induced proteoliposome fusion. We do not believe the latter to be the case because we did not detect a similar Ca^{2+}-dependent loss in channel activity using gramicidin-reconstituted liposomes (Supplementary Fig. 2**b**) suggesting that Ca^{2+} does not lead to fusion when gramicidin replaces MthK in the liposomes. To investigate whether the presence of MthK leads to Ca^{2+}-induced fusion, we also performed a contents mixing assay that did not demonstrate MthK LUV fusion (Supplementary Fig. 2**c**). We hypothesize that the lipid bilayer is a factor in MthK desensitization, because the lipid bilayer is known to be a gating modulator of ion channels[6-9].

Can MthK openings in zero Ca^{2+} explain apparent closed state block?

In order to conclude that blockers bind to closed channels in the stopped flow flux assay, it was important to demonstrate that MthK is closed in zero Ca^{2+} (Fig. 2). A very low open probability (*Po*) in zero Ca^{2+} was previously estimated using MthK recording in planar lipid bilayers (*Po* ~0.0001 to 0.001)[4]. Such a low estimate of channel activity was recapitulated using our flux assay, where our estimate of MthK *Po* was not significantly different from zero in the nominal absence of Ca^{2+} (average value of -0.00004 ± 0.00020). The estimated *Po* values were fit to a Gaussian distribution with a center at 0.0001 ± 0.0003 and standard deviation (σ) of 0.0013 ± 0.0003 (Fig. 2**e**). We therefore estimate that the *Po* is less than 0.0027 or 0.27% (the center value + 2 σ) with a probability of ~97 %. We next show that a *Po* of 0.27% is well below the MthK activity in zero Ca^{2+} that would invalidate our assumption of closed state block.

Throughout this study we interpreted the equilibration of blocker in the absence of Ca^{2+} as closed state block (Fig. 3**b**), Scheme I:

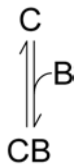

(Scheme I)

However, we can consider a situation where the blocker does not bind to the closed state but to a small fraction of channels that are open in zero Ca^{2+}. In this case, TPeA only binds to open MthK channels according to a gated access model (Scheme II). Our result that the apparent K_D^{closed} is less than K_D^{open} can be explained if the channel can close and trap the blocker inside the aqueous vestibule after it binds to the open state (see Appendix from Posson et al.[1]):

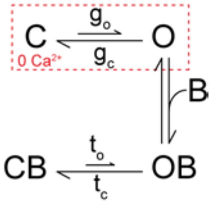

(Scheme II)

We next made the simplifying assumption that the rate of blocker trapping, t_c, is extremely high and the rate of opening with trapped blocker, t_o, is extremely low, thereby preventing blocker unbinding from the channel, Scheme III:

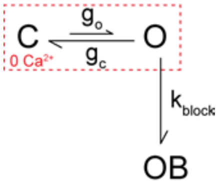

(Scheme III)

We used this simplification to calculate the blocker equilibration to the open state in such a 'worst-case scenario' for any theoretical value for channel *Po* in zero Ca^{2+} (red square in Scheme III). The state occupancies in Scheme III change in time according to:

$$\frac{d[C]}{dt} = g_c[O] - g_o[C]$$

$$\frac{d[O]}{dt} = g_o[C] - g_c[O] - k_{block}[O]$$

$$\frac{d[OB]}{dt} = k_{block}[O]$$

The equilibration of the blocked state, $[OB](t)$, is given by Eq. S1:

$$[OB](t) = 1 - A_1 e^{-\frac{t}{\tau_1}} - A_2 e^{-\frac{t}{\tau_2}} \qquad \text{(S1)}$$

The values of the exponential time constants are given by:

$$\tau_1 = \frac{2}{\sqrt{(k_{block} + g_c + g_o)^2 - 4 g_o k_{block}} + k_{block} + g_c + g_o}$$

$$\tau_2 = \frac{2}{k_{block} + g_c + g_o - \sqrt{(k_{block} + g_c + g_o)^2 - 4 g_o k_{block}}} \qquad \text{(S2)}$$

The amplitudes of the exponential components in Eq. S1, determined by the initial conditions (at t=0), $[OB]$ = 0, $[C]$ = 1-*Po*, and $[O]$ = *Po*, are given by:

$$A_1 = \frac{\tau_1 - \tau_1 \tau_2 k_{block} P_o}{\tau_1 - \tau_2}$$

$$A_2 = \frac{\tau_1 \tau_2 k_{block} P_o - \tau_2}{\tau_1 - \tau_2}$$

We numerically evaluated the parameters in $[OB](t)$, Eq. S1, using the following experimental results. From measurements of open state block (Fig. 3**e**) we estimated k_{on}^{open} = 20 μM^{-1} s^{-1} and k_{block} = 60 s^{-1} for 3 μM TPeA. Experimental recordings of single MthK channels in planar lipid bilayers determined the mean open-channel burst duration in zero Ca^{2+} to be ~100 ms[2,4], so we used for the channel closing rate, g_c = 10 s^{-1}. Since this measured burst duration was nearly independent of [Ca^{2+}], we calculated the channel opening rate for certain Po values using:

$$g_o = \frac{g_c P_o}{1-P_o}$$

Such calculations showed that for channels that are only 1% open (Po = 0.01), the time constant of blocker equilibration using this model (τ_2 = 11.6 s, Eq. S2) is 5-fold slower than our experimental results for 3 μM TPeA (τ_{eq} = 2 s, Fig. 3**b**). An equilibration time of 2 s is similar to the calculated gated access equilibration time assuming the MthK Po is ~0.1 in zero Ca^{2+}, a value which is at ~77σ for our distribution of Po estimates (Fig. 2**e**), showing that our results reflect closed state block rather than block of a small fraction of open channels in the absence of Ca^{2+}.

The above parameters were calculated using MthK gating kinetics observed in the absence of Ca^{2+} from lipid bilayer measurements[2,4]. It is possible that channel gating rates are different in the liposomal system we used in this study. If we assume a 100-fold increase in gating rate constants, g_c and g_o (Scheme III), corresponding to an open MthK burst duration of 1 ms and channel closing rate, g_c, of 1000 s^{-1}, blocker equilibration in ~2 s would require an open probability of only ~0.01, a value which is still ~7.7σ for our distribution of Po estimates (Fig. 2**e**). Further increases in gating kinetics do not cause a problem because the equilibration time constant (Eq. S2) approaches a limiting value, according to the expression:

$$\lim_{g_o \to \infty} \tau_2 = \frac{1}{k_{block} P_o}$$

Therefore, if gating is extremely fast in zero Ca^{2+}, the 2 s time constant for 3 μM TPeA equilibration could only occur in this model with a MthK Po value of 1/(60 * 2) = 0.008, a value that is ~6σ for our distribution of Po estimates (Fig. 2**e**). In conclusion, the gated access model is unable to explain the time-dependence of TPeA binding in zero Ca^{2+}, even after using extreme 'worst-case scenario' assumptions in Scheme III.

A model for state-dependent MthK block by TPeA

Here we describe a model that accounts for changes in block occurring during the stopped-flow mixing dead time. First, we assume that in the absence of Ca^{2+} blocker binds exclusively to the closed state. Second, after mixing with both Ca^{2+} and Tl^+ (at t=0), the channels immediately switch to the open state (Fig. 2**d**). Lastly, upon mixing Ca^{2+} (at t=0) the TPeA begins to re-equilibrate from closed to open state block and our measurement of the fluorescence quench rate after the mixing dead time reflects the amount of re-equilibration that occurred after 2 ms (equations S3 and S4). These assumptions reflect our inability to ascertain how activation and block evolve during the instrumental dead time. We then constrained the model using the measured open state TPeA on-rate (Fig. 3**e**, k_{on}^{open} = 20 ± 3 μM^{-1} s^{-1}) and re-estimated the closed and open state dissociation constants (K_D^{closed} = 1.3 ± 0.2 μM and K_D^{open} = 5.7 ± 0.9 μM) by fitting the closed state TPeA block dose response (Supplementary Fig. 3**a**, blue squares and blue line, Eq. S3). This lowers our estimate of K_D^{closed} by ~40% and raises our estimate of K_D^{open} by ~20%. Simulated curves for only closed or only open state binding using these model values for K_D^{closed} and K_D^{open} (dashed red and green lines, respectively, Supplementary Fig. 3**a**) illustrate how our

measurements (blue squares, Supplementary Fig. 3**a**) closely follow the red dashed line at low blocker concentrations but are deflected upwards towards the green dashed line at high blocker concentrations. We also refit the equilibration time course for 3 μM TPeA (results from Fig. 3**b**) to the model (Eq. S4, Supplementary Fig. 3**b**), which lowers our estimated closed state on-rate by ~20%, k_{on}^{closed} = 0.11 ± 0.02 μM^{-1} s^{-1} and the off-rate by ~50%, k_{off}^{closed} = 0.14 ± 0.03 s^{-1}. The resulting state-dependent block model was also used to predict the block expected from a gated access mechanism (grey dashed line, Eq. S5, Supplementary Fig. 3**a**). We note that the grey dashed line does not agree well with the measured open state block after 2 ms (grey circles) at high blocker concentrations. A re-examination of the fluorescence quenching data for these particular experiments showed that the initial decay rate was not perfectly fit by the stretched exponential function because the rapid block of open channels resulted in Tl^+ flux through channels that were far from equilibrium with the blocker. A double-exponential decay yielded a better fit the initial Tl^+ flux immediately after mixing but was more sensitive to measurement-to-measurement variability (analysis not shown). In the end, both analyses yield the same results, illustrating the robust nature of our results. The modeled TPeA state dependence is summarized in the Supplementary Table.

Modeling the Tl^+ flux rate, $k([B])$, for the closed state TPeA dose response curve, we fit the results from Fig. 3**c** to Eq. S3, using the experimental value, k_{on}^{open} = 20 μM^{-1} s^{-1}.

$$k([B]) = \frac{1}{1+\frac{[B]}{K_D^{open}}} + \left(\frac{1}{1+\frac{[B]}{K_D^{closed}}} - \frac{1}{1+\frac{[B]}{K_D^{open}}} \right) e^{-k_{on}^{open}\left([B]+K_D^{open}\right)2\,ms} \qquad \text{(S3)}$$

For modeling the closed state TPeA equilibration time dependence, $k(\delta t)$, where δt is the closed state incubation time in the delay loop, we fit the results from Fig. 3**b** to Eq. S4, using the values, k_{on}^{open} = 20 μM^{-1} s^{-1} and $[B]$ = 3 μM, as well as the values for K_D^{closed} and K_D^{open}, obtained from the fits to the dose response curve using Eq. S3 above. The free parameter in Eq. S4 is the closed state on-rate, k_{on}^{closed}.

$$k(\delta t) = \frac{1}{1+\frac{[B]}{K_D^{open}}} + \left[\frac{1}{1+\frac{[B]}{K_D^{closed}}} + \left(1 - \frac{1}{1+\frac{[B]}{K_D^{closed}}} \right) e^{-k_{on}^{closed}\left([B]+K_D^{closed}\right)\delta t} - \frac{1}{1+\frac{[B]}{K_D^{open}}} \right] e^{-k_{on}^{open}\left([B]+K_D^{open}\right)2\,ms} \qquad \text{(S4)}$$

For plotting the theoretical blocker dose response curve expected from a gated access mechanism, assuming that the blocker concentration has 2 ms of equilibration time with the open state, we used the K_D^{open} determined by equations S3 above and k_{on}^{open} = 20 μM^{-1} s^{-1} to simulate equation S5.

$$k_{gated-access}([B]) = \frac{1}{1+\frac{[B]}{K_D^{open}}} + \left(1 - \frac{1}{1+\frac{[B]}{K_D^{open}}} \right) e^{-k_{on}^{open}\left([B]+K_D^{open}\right)2\,ms} \qquad \text{(S5)}$$

Supplementary References

1 Posson, D. J., McCoy, J. G. & Nimigean, C. M. The voltage-dependent gate in MthK potassium channels is located at the selectivity filter. *Nature structural & molecular biology* **20**, 159-166, doi:10.1038/nsmb.2473 (2013).

2 Zadek, B. & Nimigean, C. M. Calcium-dependent gating of MthK, a prokaryotic potassium channel. *The Journal of general physiology* **127**, 673-685, doi:10.1085/jgp.200609534 (2006).

3 Li, Y., Berke, I., Chen, L. & Jiang, Y. Gating and inward rectifying properties of the MthK K+ channel with and without the gating ring. *The Journal of general physiology* **129**, 109-120, doi:10.1085/jgp.200609655 (2007).

4 Pau, V. P., Abarca-Heidemann, K. & Rothberg, B. S. Allosteric mechanism of Ca2+ activation and H+-inhibited gating of the MthK K+ channel. *The Journal of general physiology* **135**, 509-526, doi:10.1085/jgp.200910387 (2010).

5 Kuo, M. M., Maslennikov, I., Molden, B. & Choe, S. The desensitization gating of the MthK K+ channel is governed by its cytoplasmic amino terminus. *PLoS biology* **6**, e223, doi:10.1371/journal.pbio.0060223 (2008).

6 Yuan, C., O'Connell, R. J., Jacob, R. F., Mason, R. P. & Treistman, S. N. Regulation of the gating of BKCa channel by lipid bilayer thickness. *The Journal of biological chemistry* **282**, 7276-7286, doi:10.1074/jbc.M607593200 (2007).

7 Andersen, O. S. & Koeppe, R. E., 2nd. Bilayer thickness and membrane protein function: an energetic perspective. *Annual review of biophysics and biomolecular structure* **36**, 107-130, doi:10.1146/annurev.biophys.36.040306.132643 (2007).

8 Iwamoto, M. & Oiki, S. Amphipathic antenna of an inward rectifier K+ channel responds to changes in the inner membrane leaflet. *Proceedings of the National Academy of Sciences of the United States of America* **110**, 749-754, doi:10.1073/pnas.1217323110 (2013).

9 Rusinova, R., Kim, D. M., Nimigean, C. M. & Andersen, O. S. Regulation of ion channel function by the host lipid bilayer examined by a stopped-flow spectrofluorometric assay. *Biophysical journal* **106**, 1070-1078, doi:10.1016/j.bpj.2014.01.027 (2014).
